# Parents’ Perceived Social Support and Children’s Approaches to Learning in Rural China: A Moderated Mediation Model

**DOI:** 10.3390/ijerph192114533

**Published:** 2022-11-05

**Authors:** Zhonglian Yan, Jing Ren, Wenqi Lin, Jianfen Wu

**Affiliations:** 1 Faculty of Education, Northeast Normal University, Changchun 130024, China; 2 Jing Hengyi School of Education, Hangzhou Normal University, Hangzhou 311121, China

**Keywords:** perceived social support, approaches to learning, home learning environment, parental negative emotions, young children

## Abstract

This study aimed to investigate the relationship between parents’ perceived social support (PSS) and young children’s approaches to learning (ATLs) in rural China, as well as the mediating effect of the home learning environment (HLE) and the moderating effect of parental negative emotions (PNE). Using the cluster random sampling method, 2714 kindergarteners (*M*_age_ = 52.99 months, *SD* = 10.28; 52.00% boys; 27.43% only child) were recruited from rural areas of eight provinces in China. With questionnaires, parents reported their PSS and PNE, HLE, and the children’s ATLs. The results indicated that: (1) parents’ PSS positively predicted children’s ATLs; (2) the HLE partially mediated the relationship between parents’ PSS and children’s ATLs; and (3) PNE moderated the relationship between parents’ PSS and children’s ATLs. Thus, the results supported a moderated mediation model on the relationship between parents’ PSS and young children’s ATLs, with the HLE as a mediator and the parents’ PSS as a moderator. These findings offer new avenues for intervening and supporting the development of young children’s ATLs.

## 1. Introduction

Approaches to learning (ATLs), a core quality that influences the sustainable development of young children, is an important contributor to children’s development [1]. The ATLs is defined as the unique ways that children go about the classroom learning process and relate to how children initiate, participate in, and complete learning activities. They can be measured through various observable behaviors when they engage in learning activities, including the three dimensions of competence motivation, attention/persistence, and learning strategies [2]. Ecological systems theory suggests that young children’s development is influenced by a range of environmental factors, such as family, kindergarten, and community, and that there are interactions between these environmental factors and children’s internal factors that ultimately affect their learning and development [3]. It has been shown that parents’ PSS can significantly influence parenting levels [4], parental emotions [5], and the HLE [6]. Early parental involvement, young children’s family characteristics, and the HLE created by parents all influence the development of the ATLs in young children [7,8,9,10,11].

There is little research in the available literature on the impact of parents’ PSS on young children’s ATLs. How external environmental factors, such as family and community, contribute to young children’s ATLs is not yet clear. Meanwhile, there are some urban–rural differences in family education in China. Compared to parents in urban areas, parents in rural China possess lower economic and cultural levels, making it difficult for them to provide good family education for young children. In addition, differences in family education may contribute to differences in young children’s ATLs [12,13]. Therefore, this study recruited parents and young children in rural China as participants. Based on the ecological systems theory, the current study intends to examine how external environmental factors, both at the home level (HLE and PNE) and the community level (parents’ PSS), influence the ATLs of young children. This will not only help to clarify the role of family and community systems in the ATLs of young children in rural China but also provide directions for subsequent interventions on the ATLs of young children and for reducing the disparity between urban and rural education development in China.

### 1.1. Parents’ Perceived Social Support and Approaches to Learning in Young Children

Previous studies have shown that, in addition to internal factors such as age and temperament [14,15], external factors such as parenting style, family background characteristics, schooling style, and cultural orientation may also influence the development of the ATLs in young children to varying degrees [16,17]. Social support is the subjective support individuals receive from their social networks, the objective support they receive, and the extent to which they use the support they receive [18]. Social support generally includes family, friends, and other support [19]. Social support can effectively alleviate individuals’ psychological stress and improve their physical and mental health [20].

Prior research has shown that parents’ PSS can influence the quality of their parenting. In other words, social support can reduce parents’ stress, improve parents’ mental health and have a positive impact on parents’ parenting behavior. Parents’ mental health improves when they feel they have support and social networks at their disposal [21]. Additionally, social support can help parents regulate their emotional reactions to their children [22], and parents with higher social support levels are more consistent and caring in their parenting [23]. Sources of social support, the size of social support networks, and satisfaction with perceived and received social support can be protective factors between stress and parenting. In contrast, lacking or inadequate social support may constitute a risk factor for parental mental health, leading to inappropriate parenting practices [24]. Social support can also provide parents with information on children’s development and appropriate parenting methods, and this information and recommendations may adjust their expectations and enhance their parenting skills [25]. In other words, the higher parents’ perception of their social support level is the higher their levels of effective parenting, which are likely to play a positive role in promoting young children’s ATLs.

In China, compared with urban parents, rural parents are still in a comparatively disadvantageous situation [26]. Research indicates that children from low-income families are likely to have lower levels of the ATLs before kindergarten [27]. Educational resources in rural areas lag behind those in urban areas to varying degrees [28]. To meet their daily needs for work and family, low-income families frequently depend on their social networks for help, such as food, housing, and child care [29,30,31]. Rural and urban parents differ significantly in terms of their educational backgrounds and philosophies, how much importance they place on their children’s early education and how much time and energy they devote to it. In addition, the economic conditions of rural parents are pretty poor, and they fall behind urban parents in terms of the availability of books, products for early education, cultural activities, and interest in classes [32]. Social support plays a crucial role in parenting concepts and parents’ behavior, especially rural parents, and may affect the development of children’s ATLs.

Based on the above studies, the first hypothesis of this study is as follows:

**Hypothesis 1** **(H1).** *Parents’ perceived social support is positively related to young children’s approaches to learning.*

### 1.2. Home Learning Environment as a Mediator

There is increasing evidence that the influence of parents’ PSS on children’s physical and mental development is often mediated by various factors [21,33]. The ecosystem systems theory suggests that the external environment influences children’s physical and psychological development and that the family, the site of children’s direct activities and interactions, significantly impacts children’s development [3]. The HLE includes the range of educational activities and resources parents provide for young children. It consists of three dimensions, family learning activities, enriched life experiences, and learning materials [34]. It may play a mediating role in the relationship between parents’ PSS and young children’s ATLs.

Parents’ PSS and the HLE are closely related. It has been noted that support from family members, friends, and the community helps mothers cope with parenting stress, gain parenting skills, and provide a better home environment for young children [35,36,37]. Research indicates that the higher the level of social support, the better the HLE [6]. During the epidemic, there was a clear correlation between social support and the HLE, with higher social support associated with a better HLE [38]. Furthermore, family income was positively related to improving children’s home environment [39]. There is considerable evidence that low-income families have fewer resources that promote child development [40]. For example, impoverished children have less access to books, age-appropriate toys, etc. They are less likely to participate in learning environments outside the home, such as museums than children in nonpoor families [41]. Parents at the poverty level spend less time involved in their children’s learning activities, including less time reading to and talking with their children; they are also more likely to engage in punitive educational parenting strategies and are less likely to provide high levels of responsiveness to their young children [42,43]. Thus, rural parents at lower income levels need social support to improve their HLE.

In addition, there is a strong relationship between the HLE and the ATLs of young children. Parents and the home environment they create are children’s first teachers and classrooms. From parent–child interactions in the home and exploratory experiences inside and outside the home, children develop the fundamental social, emotional, and cognitive skills they carry into kindergarten and later schooling. The HLE is vital to children’s development [44]. Research has found that the HLE is associated with young children’s language development, reading skills, and numeracy skills [45,46,47]. The quality of the HLE is more critical for young children’s cognitive and social development than their parents’ occupation, income, and education, especially for their self-regulatory behavior, which is one of the most critical factors in children’s ATLs [48]. The HLE of African American children in Early Head Start, for example, where parents conduct home learning activities, engage in numerical and creative activities with their children, and provide learning materials, significantly and positively predicts teachers’ end-of-year ratings of young children’s ATLs [49]. Studies from China have also shown that the HLE positively predicts the ATLs of young children [11].

The second hypothesis of this study is thus that:

**Hypothesis 2** **(H2).** *The home learning environment mediates the relationship between parents’ perceived social support and young children’s approaches to learning.*

### 1.3. Parental Negative Emotions as a Moderator

Emotions can be divided into positive and negative emotions, with negative emotions underlying subjective experiences of being depressed and trapped in an unpleasant activation situation, including a variety of offensive emotional states [50], and are often measured in terms of depression, anxiety, and stress [51]. PNE may moderate the influence of parents’ PSS on the ATLs of young children. On the one hand, individuals of low socioeconomic status are more susceptible to negative emotions and have a more limited “reserve capacity” to manage resources [52,53]. Rural parents with high negative affect have difficulty using socially provided resources efficiently to support young children’s development, including the development of the ATLs. Conversely, positive emotions broaden the individual’s thinking and integrate more social and cognitive resources, making it more likely that the individual will be in good physical and mental shape and well-adjusted [54]. In this regard, parents with low negative emotions are more likely to utilize social support more efficiently to promote their young children’s development.

On the other hand, parental depression and anxiety constitute a risk for depression and anxiety in offspring [55]. Infants born to depressed or anxious parents inherit genes predisposing them to depression and anxiety. They grow up in a depressed and anxious home environment where PNE triggers insecure emotional and behavioral responses in young children [56,57,58]. When parents have high negative emotions, young children perceive PNE and develop insecurity. The effect of social support on children’s ATLs is disturbed by PNE. Therefore, this study suggests that PNE moderates the relationship between parents’ PSS and children’s ATLs.

Accordingly, hypothesis 3 is proposed as follows:

**Hypothesis 3** **(H3).** *Parental negative emotions moderate the relationship between social support and young children’s approaches to learning.*

### 1.4. The Current Study

**Figure 1:**
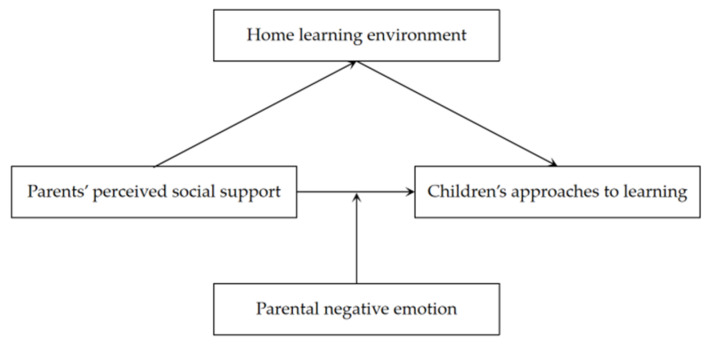
The proposed model.

Based on the above hypotheses, the current study intended to investigate the relationship between parents’ PSS and children’s approaches to learning and its underlying mechanism in rural China. We proposed a moderated mediation model (Figure 1). We attempted to answer whether (a) parents’ PSS is related to children’s ATLs, (b) the HLE mediates the relation between parents’ PSS and children’s ATLs, and (c) PNE moderates the relationship between parents’ PSS and children’s ATLs.

## 2. Materials and Methods

### 2.1. Participants

The Ethics Committee of the first author’s university approved the study. Rural parents of kindergarteners in eight provinces in China, including Jilin, Hebei, Hubei, Guangxi, and Guizhou, were surveyed by online questionnaires. The variables were reported by the parents of the children using a questionnaire. A total of 2815 questionnaires were collected. Questionnaires completed within 120 s were considered invalid. After excluding invalid questionnaires, we obtained 2714 valid data, resulting in a return rate of 96.4%. In this study, an online questionnaire was used, and submission would only be achieved with completing all questions in the questionnaire, and there were no missing values.

Among them, 82.7% of the questionnaires were completed by mothers, 16.2% by fathers, and 1.1% by other caregivers. The age distribution of children in this study was relatively even, with 34.7% of the children aged 5–6 years, 35.5% of the children aged 4–5 years, and 29.8% of the children aged 3–4 years.

### 2.2. Measures

#### 2.2.1. Perceived Social Support

The perceived social support uses a 7-point scale ranging from “strongly disagree” (1 point) to “strongly agree” (7 points), with 12 questions divided into three dimensions: family support, friend support, and other support. The original PSS has good reliability and validity [19]. The Chinese version had been translated by Jiang and Huang [59,60], and this Chinese version scale was used to measure parents’ perceived social support [61,62]. To verify the applicability of the scale in this study, we conducted an internal consistency analysis and confirmatory factors, and the results were conclusive. The Cronbach’s alpha coefficient for the scale was 0.954, and the CFA fit was good (χ^2^/df = 17.407, RMSEA = 0.078, CFI = 0.974, GFI = 0.951, AGFI = 0.917, NFI = 0.973, IFI = 0.974). The Chi-square freedom ratio (χ^2^/df) was greater than 5 because the Chi-square is influenced by the sample size; the larger the sample size is, the larger the Chi-square. Therefore, other indicators need to be combined for evaluation, and the results show that all other indicators were within the critical value range [63]. This is a appropriate scale for this study. 

#### 2.2.2. Home Observation for Measurement of the Environment

To measure the home learning environment, we used the home observation for measurement of the environment (HOME) [34], translated and used by Feng [11]. This scale is scored on a 5-point Likert scale with 20 questions divided into three dimensions: family learning activities, enriching life experiences, and learning materials. The Cronbach’s alpha coefficient for this scale was 0.918, and the CFA fit was χ^2^/df = 15.530, RMSEA = 0.073, CFI = 0.915, GFI = 0.908, AGFI = 0.883, NFI = 0.910 and IFI = 0.915. 

#### 2.2.3. Depression Anxiety Stress Scales

The depression anxiety stress scales were developed by Lovibond et al., and have been shown to have good reliability and validity [64,65]. The Chinese version of the DASS-21 has been reported for college students in mainland China, and it is suitable for adult residents in mainland China [51,66]. This study used the Chinese condensed version of the depression anxiety stress scale (DASS-21), translated by Wen et al. [66]. This questionnaire consists of 21 questions with three dimensions: depression, anxiety, and stress. In this study, a 4-point scale (0–3) was used, and the corresponding question scores for each dimension were summed and multiplied by 2 to calculate a total score for each dimension [67]. Additionally, it had good reliability in this study, with a Cronbach’s alpha coefficient of 0.955, and good CFA fits (χ^2^/df = 18.641, RMSEA = 0.081, CFI = 0.917, GFI = 0.878, AGFI = 0.845, NFI = 0.912, IFI = 0.917).

#### 2.2.4. Preschool Learning Behavior Scale

The preschool learning behavior scale (PLBS), translated and adapted by Wu et al., was used to measure the children’s ATLs [2,68]. This scale is scored on a 5-point Likert scale. It includes three dimensions: competence motivation, learning strategy, and attention. The original scale consisted of 21 questions, and during the CFA testing, it was found that some of the items on the scale were not appropriate for the analysis of this sample. Therefore, the validation factor MI was modified to remove item 12 from the “attention” dimension. After the modification, Cronbach’s alpha coefficient for this scale was 0.821, and the CFA fit was good (χ^2^/df = 12.212, RMSEA = 0.064, CFI = 0.918, GFI = 0.927, AGFI = 0.907, NFI = 0.911, IFI = 0.918).

### 2.3. Procedure

First, we established a theoretical framework for the study and constructed a moderated mediation model. Second, we invited rural parents from eight provinces in China to complete the online questionnaire. We obtained parental consent to conduct the survey, emphasized that the questionnaire was voluntary, and ensured the anonymity and confidentiality of the information collected. Third, the parents were asked to answer independently according to their actual situation. It took approximately 7 min to complete the questionnaire. Then, we inputted the collected data and filtered out invalid data.

### 2.4. Data Analysis

IBM SPSS for Windows (version 23.0, IBM, Armonk, NY, USA) was used to perform the common method deviation test, descriptive statistical analysis, and correlation analysis. AMOS 24.0 (IBM, Armonk, NY, USA) was used to perform tests and bootstrap analysis of structural equation models [69]. In this case, the bootstrap method was used to draw samples 5000 times and estimate 95% confidence intervals.

## 3. Results

### 3.1. Common Method Bias Test

This study tested the collected data for common method bias using Harman’s one-way factorial analysis [70]. The unrotated exploratory factor analysis results extracted 10 factors with characteristic roots greater than 1. The maximum factorial variance explained was 19.63%, less than the critical value of 40%, so there was no serious common method bias.

### 3.2. Descriptive Statistics and Correlation

Pearson correlation was used to evaluate the variables in this study, and the results of the descriptive statistics and correlation analysis are shown in Table 1. The results showed that the PSS of the parents of rural children, HLE, and ATLs showed a two-way correlation, and all of their dimensions showed significant positive correlations. Among the negative emotions of the rural parents of young children, depression was significantly negatively correlated with parents’ PSS (r = −0.262, *p* < 0.01), HLE (r = −0.06, *p* < 0.01), and ATLs (r = −0.327, *p* < 0.01); anxiety was significantly negatively correlated with parents’ PSS (r = −0.214, *p* < 0.01), HLE (r = −0.044, *p* < 0.05), and ATLs (r = −0.315, *p* < 0.01); and stress showed significant negative correlations with parents’ PSS (r = −0.207, *p* <0.01), HLE (r = −0.072, *p* < 0.01), and ATLs (r = −0.339, *p* < 0.01).

### 3.3. A Moderated Mediation Model

#### 3.3.1. Test of the Mediating Effect

A mediation model with the HLE as a mediator was tested using AMOS 24.0. All data were standardized before analysis, and the samples were repeated 5000 times using the bootstrap method to estimate 95% confidence intervals. Testing this mediation model showed that the mediation model fit well: χ^2^/df = 12.854, RMSEA = 0.066, CFI = 0.971, GFI = 0.976, AGFI = 0.954, NFI = 0.968, and IFI = 0.971.

Table 2 indicates that the direct effect of the parents’ PSS of rural children on the ATLs was 0.134 (95% CI (0.108, 0.165), *p* < 0.001), indicating that parents’ PSS positively predicts the ATLs. Hypothesis 1 was supported.

The indirect effect of the parents’ PSS, HLE, and ATLs was 0.037 (95% CI (0.025, 0.053), *p* < 0.001), with all confidence intervals not containing 0. The direct and indirect effects were significant, indicating that the HLE partially mediates the relationship between parents’ PSS and the ATLs. Hypothesis 2 was supported.

#### 3.3.2. Test of the Moderated Mediation Model

**Figure 2:**
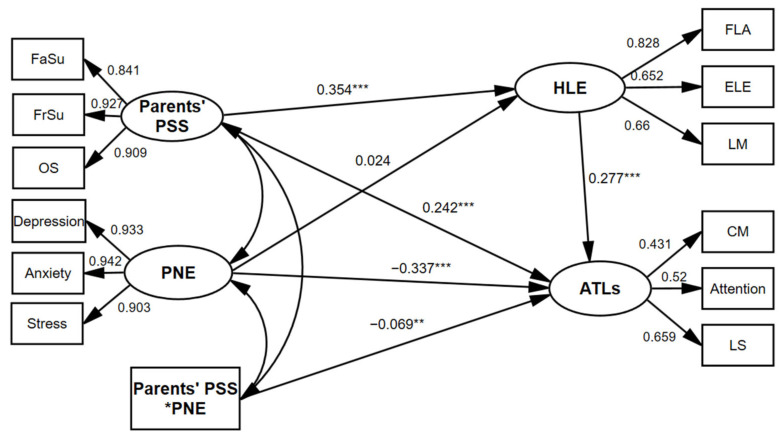
A moderated mediation model. * *p* < 0.05, ** *p* < 0.01, *** *p* < 0.001; Parents’ PSS = Parents’ perceived social support, OS = Other support, PNE = Parental negative emotion, HLE = Home learning environment, FLA = Family learning activities, ELE = Enriching life experience, LM = Learning materials, ATLs = children’s approaches to learning, CM = Competence motivation, LS = Learning strategy.

According to the research hypothesis, the latent variable structural equation model shown in Figure 2 was constructed. The model was tested based on previous studies using the same bootstrap method with 5000 replicate samples and estimated 95% confidence intervals [71]. The results show that the moderated mediating model fits well (χ^2^/df = 11.608, RMSEA = 0.063, CFI = 0.963, GFI = 0.954, AGFI = 0.931, NFI = 0.960, IFI = 0.963). PNE significantly and negatively predicted the ATLs (β = −0.337, 95% CI [−0.398, 0.277]). Meanwhile, the interaction term between parents’ PSS and PNE significantly and negatively predicted the ATLs (β = −0.077, 95% CI (−0.135, 0.016)), with confidence intervals not containing 0, which was statistically significant, demonstrating that the PNE played a moderating role on the relationship between parents’ PSS and children’s ATLs. Hypothesis 3 was supported.

**Figure 3:**
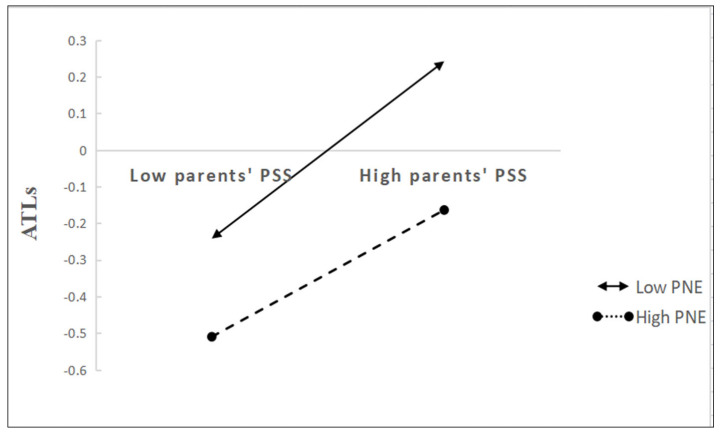
The interaction between parents’ PSS and PNE on preschool children’s ATLs. Parents’ PSS = Parents’ perceived social support, PNE = Parental negative emotion, ATLs = Children’s approaches to learning.

To more deeply explore the mechanism of the role of PNE on the relationship between parents’ PSS and ATLs, the study divided PNE into high (M+1SD) and low (M−1SD) subgroups and plotted a simple slope plot. As shown in Figure 3, for parents with low negative emotions, high parents’ PSS was significantly associated with a high level of children’s ATLs (β = 0.319, 95% CI (0.230, 0.406)). This relationship was still significant for parents with high negative emotions but much weaker (β = 0.165, 95% CI (0.081, 0.247)).

## 4. Discussion

This study examined the relationship between parents’ PSS and young children’s ATLs. We examined the mediating role of the HLE on the relationship between parents’ PSS and young children’s ALTs and the moderating role of PNE in this relationship. Our hypotheses were supported.

### 4.1. Parents’ Perceived Social Support Positively Related to Young Children’s ATLs

Our results indicated that parents’ PSS positively relates to young children’s ATLs. Parents’ mental health improves when parents feel they have support and social networks at their disposal [21]. Social support can also help parents regulate their emotional reactions to their children [22]. However, lacking social support may constitute a risk factor for parental mental health, leading to inappropriate parenting practices [24]. Meanwhile, previous research has also pointed out that parents with higher social support levels are more consistent and care about their parenting [23]. Social support can also provide parents with children’s development of information and the appropriate parenting methods, and the development of the information and recommendations may adjust their parents’ expectations and enhance their parenting skills [25]. In other words, the higher parents’ PSS level is, the higher the levels of effective parenting [4], which is likely to play a good role in promoting young children’s ATLs. The findings of this study also support the ecological systems theory that young children’s development is influenced by a range of environmental factors from their families and communities [3].

### 4.2. The Mediating Role of the Home Learning Environment

Our results suggested that the HLE partially mediates the relationship between parents’ PSS and children’s ATLs. That is, parents’ PSS can contribute partially to the development of the ATLs in children by enhancing the HLE. Social support has been shown to facilitate a good learning environment [6]. Support from family members, friends, and the community help mothers cope with parenting stress, gain parenting skills, and provide a better home environment for young children [35,36,37]. The higher the level of social support, the better the HLE [6]. Families with a good learning environment have a richer range of learning materials and can provide learning activities that are more relevant to children’s life experiences [34] in which; by participating in these activities, children’s persistence is exercised, their motivation to learn is moderately stimulated, they subsequently develop learning strategies [2], and their knowledge and skills increase [72,73]. Research has found that the HLE is associated with young children’s language development, reading skills, and numeracy skills [45,46,47]. The quality of the HLE is more critical for young children’s cognitive and social development than their parents’ occupation, income, and education, especially for their self-regulatory behavior, which is one of the most critical factors in children’s ATLs [48]. The HLE of African American children in Early Head Start, for example, where parents conduct home learning activities, engage in numerical and creative activities with their children, and provide learning materials, significantly and positively predicts teachers’ end-of-year ratings of young children’s ATLs [49]. Studies from China have also shown that the HLE positively predicts the ATLs of young children [11]. Therefore, parents’ PSS is positively related to the HLE, the HLE is positively associated with children’s ATLs, and parents’ PSS can partially promote the development of children’s ATLs by enhancing the HLE.

### 4.3. The Moderating Role of Parental Negative Emotions

This study finds that PNE moderates the relationship between rural parents’ PSS and children’s ATLs. Compared with parents with high negative emotions, low negative emotions parents’ PSS exhibited a stronger relationship with children’s ATLs. Research points out that higher levels of negativity can cause additional stress and burden that is detrimental to an individual’s healthy development [74]. Moreover, individuals with low socioeconomic status are more susceptible to negative emotions, and their “reserve capacity” to manage resources is more limited [52,53]. Conversely, positive emotions broaden the individual’s thinking and integrate more social and cognitive resources, making it more likely that the individual will be in good physical and mental shape and well-adjusted [54]. That is to say, parents with low negative emotions are more likely to use the power of social support efficiently to promote all aspects of their young children’s development. At the same time, PNE triggers children’s unsafe emotional and behavioral responses [58]. For parents with high levels of negative emotions, their children will also feel the PNE, which causes insecurity in children. Therefore, high levels of PNE negatively affect the positive relationship between parents’ PSS and children’s ATLs. In addition, this study finds that the range covers positive to negative numbers to low PNE group. It indicates that children of parents with low negative emotions had higher ALTs than the mean. Additionally, children of parents with low negative emotion had faster growth in ALTs. 

### 4.4. Limitations and Future Directions

There are certain limitations to this paper. First, the ATLs of young children in this study were evaluated by their parents. To make the assessment more accurate, teacher’s evaluation on children’s ATLs should also be collected in the future. Second, this study used cross-sectional research methods that cannot investigate causal relationships among variables. Future studies should apply longitudinal research methods to examine these relations found in the current study. Third, the HLE partially mediated the relationship between parents’ PSS and children’s ATLs. In future research, more mediating variables should be investigated to determine the mechanisms by which parents’ PSS affects children’s ATLs.

## 5. Conclusions

Notwithstanding the above limitations, to the best of our knowledge, our study is the first to explore how parents’ PSS related to young children’s ATLs from the perspective of the HLE and PNE. Our findings found that parents’ PSS was positively associated with children’s ATLs. Furthermore, the HLE mediates the relationship between parents’ PSS and children’s ATLs. PNE moderates the relationship between parents’ PSS and children’s ATLs. Specifically, for parents with higher negative emotions, the positive relationship between parents’ PSS and children’s ATLs is weaker than that of parents with lower negative emotions. 

Our findings provide a new perspective into the development mechanism of children’s ATLs, and bring some insights to family education and community education in rural China and even in less developed areas of the world. We encourage the provision of necessary social support for parents, and also encourage parents to build a good HLE to promote further development of children’s ATLs. In addition, in order to strengthen the positive impact of social support on early childhood ATLs, PNE should be reduced. In conclusion, the findings of this study provide new ways to intervene and support the development of the ATLs in young children.

## Figures and Tables

**Table 1:** Descriptive statistics and correlations among variables.

	1	2	3	4	5	6	7	8	9	10	11	12	13	14	15
1. FaSu	-														
2. FrSu	0.778 **	-													
3. OS	0.760 **	0.844 **	-												
4. FLA	0.243 **	0.249 **	0.257 **	-											
5. ELE	0.155 **	0.188 **	0.189 **	0.549 **	-										
6. LM	0.257 **	0.248 **	0.258 **	0.541 **	0.424 **	-									
7. CM	0.136 **	0.123 **	0.119 **	0.213 **	0.132 **	0.099 **	-								
8. Attention	0.312 **	0.277 **	0.280 **	0.198 **	0.087 **	0.209 **	0.082 **	-							
9. LS	0.222 **	0.191 **	0.219 **	0.167 **	0.103 **	0.167 **	0.313 **	0.386 **	-						
10. Depression	−0.279 **	−0.233 **	−0.215 **	−0.074 **	0.03	−0.056 **	−0.273 **	−0.179 **	−0.231 **	-					
11. Anxiety	−0.233 **	−0.192 **	−0.168 **	−0.061 **	0.044 *	−0.044 *	−0.273 **	−0.156 **	−0.209 **	0.878 **	-				
12. Stress	−0.223 **	−0.189 **	−0.163 **	−0.090 **	0.00014	−0.047 *	−0.326 **	−0.102 **	−0.197 **	0.841 **	0.853 **	-			
13. Parents’ PSS	0.914 **	0.941 **	0.931 **	0.268 **	0.190 **	0.274 **	0.136 **	0.313 **	0.227 **	−0.262 **	−0.214 **	−0.207 **	-		
14. HLE	0.277 **	0.283 **	0.292 **	0.916 **	0.699 **	0.800 **	0.192 **	0.217 **	0.185 **	−0.060 **	−0.044 *	−0.072 **	0.305 **	-	
15. ATLs	0.252 **	0.225 **	0.230 **	0.268 **	0.156 **	0.180 **	0.900 **	0.445 **	0.614 **	−0.327 **	−0.315 **	−0.339 **	0.254 **	0.261 **	-
*M*	5.110	4.903	4.821	2.721	2.022	2.841	3.507	3.599	3.209	5.384	5.691	8.191	4.944	2.617	3.431
*SD*	1.197	1.175	1.102	0.755	0.674	0.837	0.627	0.810	0.369	6.754	6.490	7.371	1.075	0.641	0.432

Note: * *p* < 0.05, ** *p* < 0.01; FaSu = Family Support, FrSu = Friend Support, OS = Other support, FLA = Family learning activities, ELE = Enriching life experience, LM = Learning materials, CM = Competence motivation, LS = Learning strategy, Parents’ PSS = Parents’ perceived social support (total), HLE = Home learning environment (total), ATLs = children’s approaches to learning (total).

**Table 2:** Mediating effect and 95% confidence interval estimated by the bootstrap method.

	Estimate	Bias-Corrected 95% CI			Percentile 95% CI			Percentage of Effect
		Lower	Upper	*p*	Lower	Upper	*p*	
Indirect effect	0.037	0.025	0.053	***	0.025	0.052	***	21.77%
Direct effect	0.134	0.108	0.165	***	0.105	0.162	***	78.23%
Total effect	0.172	0.140	0.208	***	0.138	0.205	***	

Note: *** *p* < 0.001.

## Data Availability

Data are available from the corresponding author upon reasonable request.

## References

[1] Chen J.-Q., Masur A., McNamee G. (2011). Young children’s approaches to learning: A sociocultural perspective. Early Child Dev. Care.

[2] McDermott P.A., Rikoon S.H., Fantuzzo J.W. (2014). Tracing children’s approaches to learning through head start, kindergarten, and first grade: Different pathways to different outcomes. J. Educ. Psychol..

[3] Bronfenbrenner U. (1979). The Ecology of Human Development: Experiments by Nature and Design.

[4] Mcconnell D., Breitkreuz R., Savage A. (2011). From financial hardship to child difficulties: Main and moderating effects of perceived social support. Child Care Health Dev..

[5] Dai Y., Fang H. (2020). Meta-analysis of the relationship between social support and mental health in Chinese. Chin. J. Health Psychol..

[6] Chang Y.E. (2017). Pathways from mothers’ early social support to children’s language development at age 3. Infant Child Dev..

[7] Buek K.W. (2019). Early growth trajectories of children’s approaches to learning: The contribution of parent and family characteristics. Psychol Schs..

[8] Bojczyk K.E., Haverback H.R., Pae H.K. (2018). Investigating maternal self-efficacy and home learning environment of families enrolled in head start. Early Child Educ. J..

[9] Leyva D., Yeomans-Maldonado G., Weiland C., Shapiro A. (2022). Latino kindergarteners’ math growth, approaches to learning, and home numeracy practices. J. Appl. Dev. Psychol..

[10] Meng C. (2015). Home literacy environment and head start children’s language development: The role of approaches to learning. Early Educ Dev..

[11] Feng L. (2020). The mediation effect of home learning environment between family socioeconomic status and preschool children’s approaches to learning. Stud. Presch. Educ..

[12] Liu C., Georgiou G.K., Manolitsis G. (2018). Modeling the relationships of parents’ expectations, family’s SES, and home literacy environment with emergent literacy skills and word reading in Chinese. Early Child Res. Q..

[13] Rao N., Sun J., Ng M., Becher Y., Lee D., Ip P., Bacon-Shone J. (2014). Validation, Finalization and Adoption of the East Asia-Pacific Early Child Development Scales (EAP-ECDS).

[14] Kagan S.L., Moore E.K., Bredekamp S. (1995). Reconsidering Children’s Early Development and Learning: Toward Common Views and Vocabulary.

[15] Domínguez X., Vitiello V.E., Fuccillo J.M., Greenfield D.B., Bulotsky-Shearer R.J. (2011). The role of context in preschool learning: A multilevel examination of the contribution of context-specific problem behaviors and classroom process quality to low-income children’s approaches to learning. J. Sch. Psychol..

[16] Burchinal M.R., Peisner-Feinberg E., Pianta R., Howes C. (2002). Development of academic skills from preschool through second grade: Family and classroom predictors of developmental trajectories. J. Sch. Psychol..

[17] Williams N.C. (2002). The relationship of home environment and kindergarten readiness. Ph.D. Thesis.

[18] Xiao S. (1994). Theoretical foundations and research applications of the Social Support Scale. J. Clin. Psychol. Med..

[19] Zimet G.D., Dahlem N.W., Zimet S.G., Farley G.K. (1988). The multidimensional scale of perceived social support. J. Pers. Assess..

[20] Cohen S., McKay G. (2020). Social support, stress and the buffering hypothesis: A theoretical analysis. Handbook of Psychology and Health.

[21] Taylor Z.E., Conger R.D., Robins R.W., Widaman K.F. (2015). Parenting practices and perceived social support: Longitudinal relations with the social competence of Mexican-origin children. J. Lat. Psychol..

[22] Marroquin B. (2011). Interpersonal emotion regulation as a mechanism of social support in depression. Clin. Psychol. Rev..

[23] Byrnes H.F., Miller B.A. (2012). The relationship between neighborhood characteristics and effective parenting behaviors: The role of social support. J. Fam. Issues.

[24] Belsky J., Jafee S.R., Cicchetti D., Cohen D.J. (2015). The multiple determinants of parenting. Developmental Psychopathology: Risk, Disorder, and Adaptation.

[25] Cochran M., Niegro S., Bornstein M.H. (2002). Parenting and social networks. Handbook of Parenting: Social Conditions and Applied Parenting.

[26] Gao S., Bian Y. (2022). Construction strategy of family education guidance service system under the background of rural revitalization. Res. Educ. Dev..

[27] Gullo D.F. (2017). A structural model of early indicators of school readiness among children of poverty. J. Child. Poverty.

[28] Yu S., Liu F. (2021). Evolution and development prospect of rural education from the perspective of urban-rural relations. Res. Educ. Dev..

[29] Heflin C., London A.S., Scott E.K. (2011). Mitigating material hardship: The strategies low-income families employ to reduce the consequences of poverty. Sociol. Inq..

[30] Conrad S. (1997). Making ends meet: How single mothers survive welfare and low-wage work. J. Public Health Pol..

[31] Henly J.R., Danziger S.K., Offer S. (2005). The contribution of social support to the material well-being of low-income families. J. Marriage Fam..

[32] Chen Q. (2022). Complexity challenge and path of the balanced development of urban and rural pre-school education in the new era base on the theory of complexity. Soc. Sci. Front..

[33] Lu M., Chen J., He W., Pang F., Zou Y. (2021). Association between perceived social support of parents and emotional/behavioral problems in children with ASD: A chain mediation model. Res. Dev. Disabil..

[34] Totsika V., Sylva K. (2004). The home observation for measurement of the environment revisited. Child Adolesc. Ment. Health.

[35] Koeske G.F., Koeske R.D. (1990). The buffering effect of social support on parental stress. Amer. J. Orthopsychiat..

[36] Warren P.L. (2005). First-time mothers: Social support and confidence in infant care. J. Adv. Nurs..

[37] Burchinal M., Follmer A., Bryant D. (1996). The relations of maternal social support and family structure with maternal responsiveness and child outcomes among African-American families. Dev. Psychol..

[38] Zhang C., Qiu W., Li H., Li J., Zhang L., Li X., Li J. (2021). Parental stress and home activities for young children during the stay-at-home quarantine time in China. Early Educ. Dev..

[39] Dearing E., Taylor B.A. (2007). Home improvements: Within-family associations between income and the quality of children’s home environments. J. Appl. Dev. Psychol..

[40] Evans G.W. (2004). The environment of childhood poverty. Am. Psychol..

[41] Bradley R.H., Corwyn R.F., McAdoo H.P., Coll C.G. (2001). The home environments of children in the United States: Part I. Variations by age, ethnicity, and poverty status. Child Dev..

[42] Hoff E. (2003). The specificity of environmental influence: Socioeconomic status affects early vocabulary development via maternal speech. Child Dev..

[43] McLeod J.D., Shanahan M.J. (1993). Poverty, parenting, and children’s mental health. Am. Sociol. Rev..

[44] Chazan-Cohen R., Raikes H., Brooks-Gunn J., Ayoub C., Pan B.A., Kisker E.E., Roggman L., Fuligni A.S. (2009). Low-income children’s school readiness: Parent contributions over the first five years. Early Educ. Dev..

[45] Murray A.D., Yingling J.L. (2000). Competence in language at 24 months: Relations with attachment security and home stimulation. J. Genet. Psychol..

[46] Morrison F.J., Cooney R.R., Borkowski J.G., Ramey S.L., Bristol-Power M. (2001). Parenting and academic achievement: Multiple paths to early literacy. Parenting and the Child’s World: Influences on Academic, Intellectual, and Socioemotional Development.

[47] Melhuish E.C., Phan M.B., Sylva K., Sammons P., Siraj-Blatchford I., Taggart B. (2008). Effects of the home learning environment and preschool center experience upon literacy and numeracy development in early primary school. J. Soc. Issues.

[48] Pam S., Karen E., Kathy S., Edward M., Iram S., Brenda T. (2004). The impact of pre-school on young children’s cognitive attainments at entry to reception. Br. Educ. Res. J..

[49] Fantuzzo J., Tighe E., Childs S. (2000). Family involvement questionnaire: A multivariate assessment of family participation in early childhood education. J. Educ. Psychol..

[50] Li X., Luo J., Gao W., Yuan J. (2009). Research on negative emotions, coping style, self-esteem and interpersonal relationship of college students with left-behind experience. Chinese J. Clin. Psychol..

[51] Gong X., Xie X., Xu R., Luo Y. (2010). Psychometric properties of the Chinese versions of DASS-21 in Chinese college students. Chinese J. Clin. Psychol..

[52] Shelley T., Teresa E.S. (1999). Psychosocial resources and the SES-health relationship. Ann. N. Y. Acad. Sci..

[53] Linda C.G., Karen A.M. (1999). Do negative emotions mediate the association between socioeconomic status and health?. Ann. N. Y. Acad. Sci..

[54] Tammy J.C., Jessica L.A. (2014). Review of the handbook of positive emotions. J. Soc. Psychol..

[55] Beardslee W.R., Gladstone T.R., O’Connor E.E. (2011). Transmission and prevention of mood disorders among children of affectively ill parents: A review. J. Am. Acad. Child Psy..

[56] Eley T.C., McAdams T.A., Rijsdijk F.V., Lichtenstein P., Narusyte J., Reiss D., Spotts E.L., Ganiban J.M., Neiderhiser J.M. (2015). The intergenerational transmission of anxiety: A children-of-twins study. Am. J. Psychiatry.

[57] Nivard M.G., Dolan C.V., Kendler K.S., Kan K.J., Willemsen G., van Beijsterveldt C.E.M., Lindauer R.J.L., van Beek J.H.D.A., Geels L.M., Bartels M. (2015). Stability in symptoms of anxiety and depression as a function of genotype and environment: A longitudinal twin study from ages 3 to 63 years. Psychol. Med..

[58] Cummings E.M., Goeke-Morey M.C., Papp L.M., Dukewich T.L. (2002). Children’s responses to mothers’ and fathers’ emotionality and tactics in marital conflict in the home. J. Fam. Psychol..

[59] Jiang Q., Guo H., Chen D., Chen Y., Wu J., Zhao S. (1998). Medical Psychology.

[60] Huang L., Jiang Q., Ren W. (1996). Research on the correlation between coping style, social support, and psychosomatic symptoms of cancer patients. Chin. Ment. Health J..

[61] Gao L.L., Sun K., Chan S.W.C. (2014). Social support and parenting self-efficacy among Chinese women in the perinatal period. Midwifery.

[62] Zhao L., Wang Y., Bai J., Lei X. (2020). Rumination and anxiety: The mediating role of perceived social support. China J. Health Psychol..

[63] Wu M. (2009). Structural Equation Modeling—Operation and Application of AMOS.

[64] Lovibond P.F., Lovibond S.H. (1995). The structure of negative emotional states: Comparison of the Depression Anxiety Stress Scales (DASS) with the beck depression and anxiety inventories. Behav. Res. Ther..

[65] Antony M.M., Cox B.J., Enns M.W., Bieling P.J., Swinson R.P. (1998). Psychometric properties of the 42-item and 21-item versions of the Depression Anxiety Stress Scales in clinical groups and a community sample. Psychol. Assess.

[66] Wen Y., Wu D., Lv X., Li H., Liu X., Yang Y., Xu Y., Zhao Y. (2012). Psychometric properties of the Chinese short version of depression anxiety and stress scale in Chinese adults. China J. Health Psychol..

[67] Chew N., Lee G., Tan B., Jing M., Goh Y., Ngiam N., Yeo L., Ahmad A., Khan F., Shanmugam G. (2020). A Multinational, Multicentre Study on the Psychological Outcomes and Associated Physical Symptoms Amongst Healthcare Workers During COVID-19 Outbreak. Brain Behav. Immun..

[68] Wu Z., Hu B.Y., Fan X. (2019). Cross-cultural validity of preschool learning behavior scale in Chinese cultural context. J. Psychoeduc. Assess..

[69] Wen Z., Ye B. (2014). Analyses of Mediating Effects: The Development of Methods and Models. Adv. Psychol Sci.

[70] Zhou H., Long L. (2004). Statistical remedies for common method biases. Adv. Psychol. Sci..

[71] Wen Z., Ye B. (2014). Different methods for testing moderated mediation models: Competitors or backups?. Acta. Psychol. Sin..

[72] Stingone J.A., Claudio L. (2017). The combined influence of air pollution and home learning environment on early cognitive skills in children. Int. J. Environ. Res. Public Health.

[73] Anders Y., Rossbach H.G., Weinert S., Ebert S., Kuger S., Lehrl S., von Maurice J. (2012). Home and preschool learning environments and their relations to the development of early numeracy skills. Early Child Res. Q.

[74] Fischer A.H. (2018). Comment: The emotional basis of toxic affect. Emot. Rev..

